# Calciphylaxis in end-stage kidney disease: outcome data from the United Kingdom Calciphylaxis Study

**DOI:** 10.1007/s40620-020-00908-9

**Published:** 2021-02-06

**Authors:** Rajkumar Chinnadurai, Abby Huckle, Janet Hegarty, Philip A Kalra, Smeeta Sinha

**Affiliations:** 1 Department of Renal Medicine, Salford Royal NHS Foundation Trust, Salford, M6 8HD UK; 2 Faculty of Biology, Medicine and Health, University of Manchester, Manchester, UK

**Keywords:** Calciphylaxis, End-stage kidney disease, Haemodialysis, Mortality

## Abstract

**Background and aims:**

Calciphylaxis is a rare condition associated with very high mortality in patients with end-stage kidney disease. Data from country-based registries have been an invaluable resource for a better understanding of the natural history and management for this condition. This study aimed to investigate the current management strategies and outcomes of patients enrolled in the United Kingdom Calciphylaxis study (UKCS).

**Methods:**

The study was conducted on 89 patients registered in the UKCS since 2012. The initial analysis included a description of the baseline characteristics, management strategies and outcomes on follow-up until May 2020. Further analysis included a comparison of the mortality outcome of the UKCS patients who were receiving haemodialysis with a propensity score matched cohort of haemodialysis patients from the Chronic Renal Insufficiency Standards Implementation Study- Haemodialysis (CRISIS-HD).

**Results:**

Median age of the cohort was 59 years, with a predominance of females (61%) and Caucasian (95%) ethnicity. About 54% of the patients were diabetic and 70% were receiving haemodialysis at study entry. The skin lesions were mostly distributed in the lower extremities (48%). Sodium thiosulphate and calcimimetic were the most widely used management strategies. The mortality rate was 72 deaths per hundred patient-years (50 deaths observed in 69.5 patient years). Complete wound healing was noted in 17% and bacteraemia was reported in 26% of patients. In a comparative analysis of the matched haemodialysis patients, the presence of calciphylaxis in 62 patients showed a strong association with all-cause mortality (HR 6.96; p < 0.001), with annual mortality 67% versus 10.2% in haemodialysis patients without calciphylaxis.

**Conclusions:**

This UK wide study strengthens the evidence that calciphylaxis is a strong and independent risk factor associated with all-cause mortality; no significant benefit was shown with any individual treatment modality. Until further evidence becomes available, a multifaceted approach would be the appropriate treatment strategy in the management of this extremely serious condition.

**Graphic abstract:**

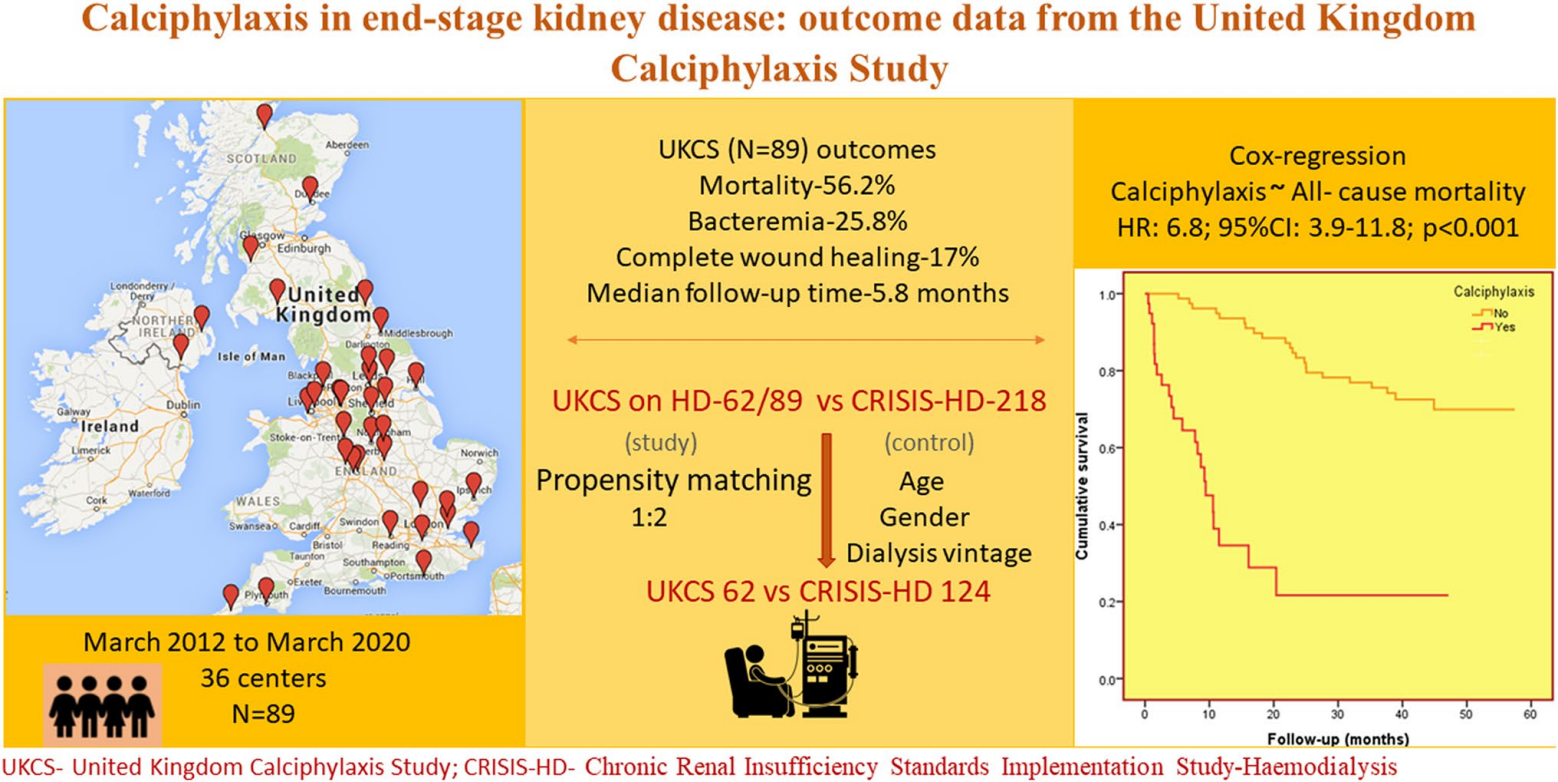

**Electronic supplementary material:**

The online version of this article (10.1007/s40620-020-00908-9) contains supplementary material, which is available to authorized users.

## Introduction

Calciphylaxis, or calcific uremic arteriolopathy (CUA), is a rare but deleterious condition in which small arterioles within the skin become calcified. This in turn results in classically painful skin lesions, particularly in patients with end-stage kidney disease (ESKD). The reported prevalence of CUA in dialysis patients ranges from 1–4%, with an annual incidence of around 0.04% [1, 2]. The annual mortality rate is reported to be as high as 60% in patients with CUA [3]. Despite the high mortality rate, there are no diagnostic tests or licensed treatments for the condition. Researchers have, however, identified potential risk factors that may be associated with worse outcomes in CUA. Risk factors including cardiovascular disease, diabetes mellitus and warfarin use have been associated with all-cause mortality in patients with CUA possibly due to co-existing systemic vascular calcification [4]. The management of CUA remains problematic as clinicians have largely relied on case series and small uncontrolled studies for guidance. Several agents such as sodium thiosulphate, calcimimetics [5], bisphosphonates [6] and vitamin K have been identified as showing promise in small studies, although real benefit of these agents in isolation is still under debate [7–9]. Country-based registries and studies have been a major resource in providing data on the natural history and outcomes of CUA [10]. Ongoing evaluation of real-world management strategies and outcomes are warranted for optimising care in this poorly understood condition.

This study aims to investigate the management strategies and outcomes of patients with CUA registered in the United Kingdom (UK) calciphylaxis study. Adding to this, the study also compares the outcomes and the strength of association of calciphylaxis in haemodialysis patients with a matched group of patients who received haemodialysis but without development of calciphylaxis.

## Methods

The United Kingdom Calciphylaxis Study (UKCS) is an ongoing nationwide prospective observational cohort study. The first patient was recruited to the study in 2012; details of patient recruitment is described online at www.calciphylaxis.org.uk. Currently around 36 renal centres across the UK have recruited patients to the study. In brief, any chronic kidney disease patient, including patients with ESKD receiving dialysis, diagnosed with calciphylaxis from any UK renal department is approached to be consented and recruited to this study at the time of diagnosis. At study baseline (consent date), details including demographics, concomitant medications, standard laboratory variables and clinical information on skin lesions, symptoms, and initial therapeutic interventions is collected. In addition, blood samples for DNA analysis, plasma/serum for biomarkers and clotting samples are collected and stored at − 80 °C for future analysis. Follow-up clinical and laboratory data is collected every four months for all patients until the study endpoints of complete wound healing or death. The study gained ethical approval from the National Research Ethics Service (reference: 11/NW/0528).

**Fig. 1:**
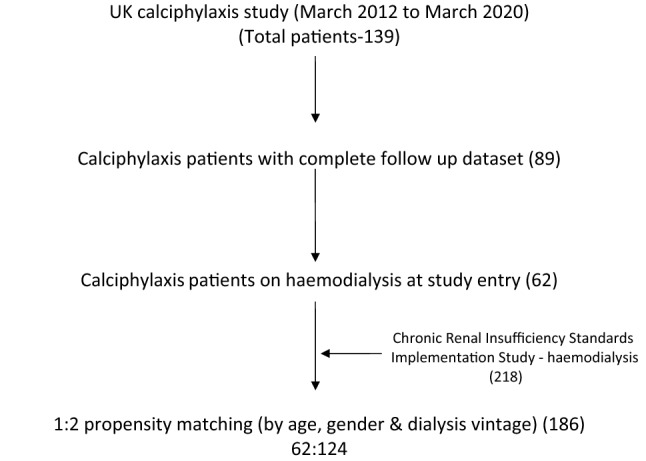
Flowchart of patient sampling

An initial cross-sectional analysis was carried out on 89 patients with complete follow-up datasets in the UKCS between June 2013 and May 2020. The analysis included a description of baseline characteristics, management strategies and outcomes. Of the 89 patients, 62 were receiving haemodialysis at UKCS entry. These 62 patients were separately considered with a propensity matched group from the Chronic Renal Insufficiency Standards Implementation Study-Haemodialysis (CRISIS-HD) database for a comparative analysis (Fig. 1).

The CRISIS-HD was a prospective study which recruited haemodialysis patients between March 2012 and 2014, and is now subsumed into the Salford Kidney Study. Patient recruitment into CRISIS-HD is described in published literature [11, 12]. In summary, patients aged > 18 years old receiving maintenance haemodialysis at Salford Royal hospital and its four satellite units (a tertiary renal centre in the United Kingdom with a catchment population of 1.55 million) were approached to be consented for participation in this study. The exclusion criteria were the inability to participate due to illness or poor mobility and incapacity to consent; a total of 218 patients were recruited into CRISIS-HD.

At CRISIS-HD study baseline (consent date), data including demographics, comorbidities, laboratory results and three month-averaged dialysis details were recorded from electronic patient records (EPR). All CRISIS-HD patients were followed up until the study endpoints which included renal transplantation, death, and the study end date of 31 December 2016. Transplantation and mortality records were gathered from EPR. The CRISIS-HD adhered to the Declaration of Helsinki and has ethical approval for all observational data (REC 05/Q1404/188).

The 62 UKCS patients were matched for age, gender and dialysis vintage (years receiving dialysis) with the 218 patients in the CRISIS-HD cohort by propensity score matching. Matching was undertaken by 1:2 neighbour matching of patients with the same propensity scores as generated by the R software (version 3.5.1) `MatchIt’ package [13, 14] (Supplementary Fig. 1). The resultant matched cohort of 186 patients were used for comparative analysis. In the descriptive analysis of the comparative data, the Mann-Whitney U test was used to generate p-values for continuous data and the Chi-square test was used for obtaining p-values for categorical data. Univariable and multivariable Cox-regression analysis was used to study the strength of the association between the presence of calciphylaxis and all-cause mortality. Multivariable models were developed by adjusting for factors that were statistically significant in the univariable model. Due to the small number of events, a maximum of six variables were included in each of the multivariable models to preserve stability [15]. The proportional hazard assumption was examined and met by plotting the log-minus-log survival curves and survival times against cumulative survival. Kaplan-Meier survival curves were used to illustrate cumulative survival between the groups. All analysis was performed using SPSS (version 22), licenced to the University of Manchester.

## Results

### United Kingdom Calciphylaxis Study (UKCS) outcomes

The median age of the 89 patients in the UKCS cohort was 59 years with a predominance of females (61%) and Caucasians (95%). At study entry a majority (70%) were receiving haemodialysis, 18% were receiving peritoneal dialysis, and 12% were non-dialysis chronic kidney disease patients. The diagnosis of calciphylaxis was made by clinical impression in 52 (58.5%) patients, by clinical impression and radiology of soft tissue in 5 (5.5%) patients, and by skin biopsy in 33 (37%) patients. Fifty-four per cent were diabetic, with a median body mass index of the cohort being 31 kg/m^2^. Vitamin D analogues were prescribed for 68%, with 41% receiving a vitamin K antagonist. The skin lesions were predominantly distributed in the lower extremities (49%) followed by the abdomen (30%) and thighs (25%). The median haemoglobin and albumin values were reduced compared to the normal range values; however, the c-reactive protein was elevated (Table 1). A comparison of baseline characteristics between the patients who had complete wound healing and those who did not showed a statistically significant higher haemoglobin and serum albumin and a lower c-reactive protein in the healed group (supplementary Table 1).

**Table 1:** Baseline characteristics of the UKCS patients

Variable	Total-89
Age, years	59 (53–69)
Gender, female	54 (60.6%)
Ethnicity, Caucasian	84 (94.4%)
Body mass index, (Kg/m^2^)	31 (25–38)
Renal status at diagnosis	
Non-dialysis chronic kidney disease	11 (12.4%)
Haemodialysis	62 (69.6%)
Peritoneal dialysis	16 (18.0%)
Co-morbidities	
Ischaemic heart disease	35 (39.3%)
Myocardial infarction	20 (22.5%)
Cerebrovascular disease	12 (13.5%)
Peripheral vascular disease	14 (15.7%)
Diabetes mellitus	48 (53.9%)
Bone fractures	2 (2.25%)
Hypertension	58 (65.2%)
Parathyroidectomy	8 (8.9%)
Medications	
Vitamin D analogues	40 (44.9%)
Calcium based phosphate binders	14 (15.7%)
Calcimimetics	17 (19.1%)
Vitamin K antagonist (warfarin)	24 (26.9%)
Distribution of skin lesions	
Abdomen	24 (26.9%)
Breast	6 (6.7%)
Lower extremities (leg/calf/toes)	43 (48.3%)
Thighs	22 (24.7%)
Buttock	8 (9%)
Groins/genitalia	4 (4.5%)
Hands and fingers	6 (6.7%)
Laboratory results	
Haemoglobin, g/L	94 (82–111)
Albumin, g/L	27 (22–35)
Corrected calcium, mmol/L	2.39 (2.24–2.53)
Phosphate, mmol/L	1.66 (1.13–2.01)
Alkaline phosphatase, units/L	157 (104–269)
Parathyroid hormone^a^, ng/L	329.5 (78–679)
C-reactive protein^b^, mg/L	65 (14–145)

Categorical variables expressed as number (percentage) and continuous variables expressed as median (interquartile range)

*UKCS* United Kingdom calciphylaxis study

^a^Missing Parathyroid hormone levels in 32 patients

^b^Missing CRP in 7 patients

The median duration of follow-up was 5.8 months; 17% of patients had complete wound healing, and 56.2% of the cohort died during the study period (June 2013 and May 2020). The mortality rate was 72 deaths per hundred patient-years (50 deaths observed in 69.5 patient-years). The mortality rate of the non-dialysis CKD patients with calciphylaxis (11 patients at recruitment) was 82 deaths per hundred patient-years (8 deaths observed in 9.75 patient-years), although four patients were commenced on dialysis after study entry. The median time for wound healing after study entry was eight months, with the median survival time being four months (Table 2).

**Table 2:** Outcomes from the UKCS patients

Outcome	Results in 89 patients
Follow-up, months	5.8 (2–11.6)
Bacteraemia	23 (25.8%)
Complete wound healing	15 (16.8%)
Time for wound healing, months	8 (3–23)
Total death	50 (56.2%)
Survival time, months	4 (1–9)

Categorical variables expressed as number (percentage) and continuous variables expressed as median (interquartile range)

*UKCS* United Kingdom calciphylaxis study

The various strategies used in the management of calciphylaxis are listed in Table 3. More than 65% of patients received sodium thiosulphate, followed by calcimimetic use in 41% specifically as a medical intervention for the treatment of calciphylaxis. Other approaches included increased dialysis frequency, surgical wound care and cessation of calcium binders, warfarin and vitamin-D analogues. The treatment approaches were not protocolled by the study and there was variation in practice across the centres. In addition to systemic treatments, local wound treatments described included wound debridement (20%) and skin grafting (3.5%).

**Table 3:** Management strategies used specifically for treatment of calciphylaxis

Management strategies	Total-89 number (%)
Calcimimetics (cinacalcet)	36 (40.5%)
Sodium thiosulphate	59 (66.3%)
Increased dialysis frequency	17 (19.1%)
Dialysis calcium concentration reduced	20 (22.5%)
Wound debridement	18 (20.2%)
Stopping calcium binders	20 (22.5%)
Stopping/reducing vitamin-D analogues	23 (25.8%)
Stopping warfarin	12 (13.4%)
Bisphosphonates	4 (4.5%)
Antibiotics (intravenous)	27 (30.3%)
Antibiotics (oral)	14 (15.7%)
Hyperbaric oxygen	4 (4.5%)
Skin graft	3 (3.5%)
Amputation	3 (3.5%)

*UKCS* United Kingdom calciphylaxis study, p-value by chi-square test

## UKCS and Salford Kidney Study-Haemodialysis (CRISIS-HD) matched analysis

The groups were well matched for age, gender and dialysis vintage after propensity score matching. A higher prevalence of diabetes was observed in the UKCS group (48% vs. 37%, p = 0.14), although the difference was not statistically significant. Patients in the UKCS group had a higher median body mass index (32 vs. 27 kg/m^2^, p-value = 0.001) and they had a higher prevalence of cardiovascular disease: history of ischemic heart disease, myocardial infarctions and peripheral vascular disease. A significantly higher proportion of deaths were noted in the UKCS group (53% vs. 31%, p-value = 0.004) resulting in a shorter median follow up period (7 vs. 39 months, p-value < 0.001). The age at death was much lower in the UKCS cohort (59 vs. 72 years, p = 0.001). The UKCS cohort had much higher mortality rate; 67 deaths per hundred patient-years (33 deaths observed in 49.6 patient-years), compared to the CRISIS-HD cohort; 10.2 deaths per hundred patient-years (39 deaths observed in 382.6 patient-years). The UKCS patients had a significantly lower haemoglobin, albumin and a higher c-reactive protein. The median parathyroid hormone level was higher in the UKCS cohort, although this was statistically different (326 vs. 200 ng/L, p = 0.29). There was a statistically significant difference between some prescribed medications at study entry, with a higher proportion on erythropoietin stimulating agents and vitamin K antagonists in the UKCS cohort (p < 0.001 for both the drugs) (Table 4).

**Table 4:** Comparison of baseline characteristics and follow-up events between UKCS and CRISIS-HD cohort in a matched sample

	Matched sample (186)		
Variables	UKCS (62)	CRISIS-HD (124)	p-value
Baseline			
Age, years	59 (53–70)	63 (51–72)	0.39
Gender, female	39 (63%)	69 (56.5%)	0.34
Ethnicity, Caucasian	59 (95.1%)	101 (81.5%)	**0.01**
Body mass index^a^, Kg/m^2^	32 (25–38)	27 (23.1–30.6)	**0.001**
Diabetes mellitus	30 (48.4%)	46 (37.1%)	**0.14**
Ischemic heart disease	25 (40.3%)	21 (16.9%)	**< 0.001**
Myocardial infarctions	14 (22.5%)	9 (7.3%)	**0.003**
Peripheral vascular disease	11 (17.7%)	9 (7.3%)	**0.03**
Cerebrovascular accident	9 (14.5%)	8 (6.5%)	0.07
Dialysis vintage, months	54 (17–100)	38 (11–81)	0.17
Corrected calcium, mmol/L	2.32 (2.2–2.5)	2.34 (2.3–2.45)	0.43
Phosphate, mmol/L	1.64 (1.12–1.85)	1.51 (1.08–1.80)	0.34
Parathyroid hormone^b^, ng/L	326 (76–695)	200 (114–441)	0.29
Albumin, g/L	29 (23–35)	39 (35–41)	**< 0.001**
Haemoglobin, g/L	94 (82–107)	107 (99–116)	**< 0.001**
C-reactive protein^c^, mg/L	41 (10–116)	8.2 (3.3–20)	**< 0.001**
Erythropoietin stimulating agents	39 (62.9%)	29 (23.4%)	**< 0.001**
Vitamin D analogue	37 (59.7%)	62 (50%)	0.212
Calcium containing phosphate binder	10 (16.1%)	30 (24.2%)	0.21
Calcimimetic (cinacalcet)	12 (19.3%)	13 (10.5%)	0.09
Vitamin K antagonist (warfarin)	22 (35.5%)	9 (7.3%)	**< 0.001**
Follow-up			
Follow-up, months	6.8 (2.0-11.5)	39 (32–45)	**< 0.001**
Total deaths	33 (53.2%)	39 (31.5%)	**0.004**
Age at death, years	59 (53–69)	72 (65–79)	**< 0.001**

Continuous data median (inter quartile range) with p-value by Mann-Whitney U test. Categorical data expressed as number (percentage) with Chi-square test for p-value

*UKCS* United Kingdom calciphylaxis study, *CRISIS-HD *Chronic Renal Insufficiency Standards Implementation Study-Haemodialysis

^a^Missing body mass index values for 11 patients in the UKCS group

^b^Missing parathyroid hormone levels in 23/62 of UKCS group and 41/124 of CRISIS-HD group

^c^Missing c-reactive protein for 3 patients in the calciphylaxis study group

**Fig. 2:**
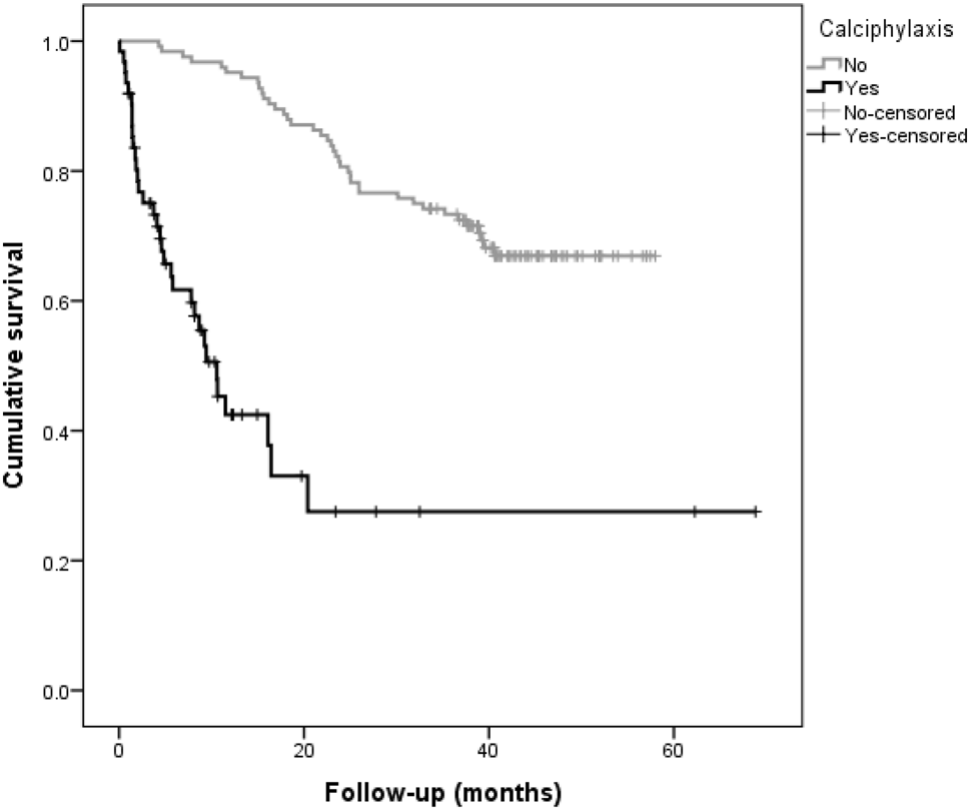
Kaplan–Meier survival curve for presence of calciphylaxis in the matched dialysis populations

In a univariable cox-regression model, the presence of CUA showed a very strong association with all-cause mortality (HR: 6.96; 95% CI 4.1–11.7; p < 0.001) (Table 5). A history of myocardial infarction was also found to be significantly associated with all-cause mortality in this model (HR: 2.76; 95% CI 1.54–4.98; p = 0.001). The strength of association of CUA extended to the multivariable model for mortality which included factors that were significant in the univariable model (HR: 6.8; 95% CI 3.9–11.8; p < 0.001) (Table 6). Other factors including older age, Caucasian ethnicity, diabetes, lower haemoglobin and albumin were noted to be strongly associated with all-cause mortality. The Kaplan-Meier plot demonstrates a significantly worse survival in patients with CUA (log-rank, p-value < 0.001) (Fig. 2).

**Table 5:** Association of calciphylaxis with all-cause mortality (Cox-regression analysis-univariable model)

Variable	HR (95% CI)	p-value
Calciphylaxis	6.96 (4.1–11.7)	**< 0.001**
Age	1.03 (1.01–1.05)	**0.002**
Male	1.16 (0.73–1.83)	0.54
Caucasian	3.7 (1.4–10.4)	**0.01**
Diabetes mellitus	1.79 (1.1–2.9)	**0.013**
BMI	1.01 (0.97–1.04)	0.86
IHD	1.61 (0.96–2.68)	0.06
MI	2.76 (1.54–4.98)	0.001
PVD	1.88 (0.99–3.57)	0.054
CVA	0.96 (0.39–2.38)	0.93
Dialysis vintage	0.99 (0.99–1.01)	0.36
Haemoglobin	0.96 (0.94–0.97)	**< 0.001**
Albumin	0.89 (0.87–92)	**< 0.001**
C-reactive protein	1.02 (1.01–1.02)	**< 0.001**
Corrected calcium	1.55 (0.41–5.77)	0.51
Phosphate	0.97 (0.63–1.46)	0.83
Parathyroid hormone	1.00 (1.00–1.01)	**0.001**
ESA	1.66 (1.03–2.67)	**0.04**
Vitamin D analogue	1.24 (0.78–1.97)	0.36
Phosphate binder	0.78 (0.44–1.39)	0.40
Calcimimetic	1.09 (0.57–2.08)	0.78
Vitamin K antagonist (warfarin)	2.67 (1.55–4.6)	**< 0.001**

Hazard ratio for Parathyroid hormone included only 122 patients with available parathyroid hormone levels

*BMI *Body mass index, *IHD *ischemic heart disease, *MI *myocardial infarction, *PVD *peripheral vascular disease, *CVA *cerebrovascular accident, *ESA *erythropoietin stimulating agent, *HR *hazard ratio, *CI *confidence interval

**Table 6:** Association of calciphylaxis with all-cause mortality (Cox-regression analysis-multivariable models 1,2,3)

	Model-1		Model-2		Model-3	
Variable	HR (95% CI)	p-value	HR (95% CI)	p-value	HR (95% CI)	p-value
Calciphylaxis	6.8 (3.9–11.8)	**< 0.001**	7.04 (3.9–12.7)	**< 0.001**	3.78 (2.02–7.11)	**< 0.001**
Age	1.03 (1.01–1.05)	**0.003**	1.03 (1.01–1.05)	**0.002**	1.03 (1.01–1.05)	**0.001**
Ethnicity	3.2 (1.16–9.1)	**0.025**	2.9 (1.06–8.2)	**0.038**	2.5 (0.89–7.1)	0.082
Diabetes mellitus	1.72 (1.08–2.74)	**0.02**	1.75 (1.09–2.8)	**0.02**	1.69 (1.05–2.73)	**0.029**
Myocardial infarction	1.78 (0.97–3.2)	0.06				
Warfarin use			1.11 (0.60–2.05)	0.73		
Haemoglobin					0.97 (0.96–0.99)	**0.015**
Albumin					0.93 (0.90–0.96)	**< 0.001**

Multivariate Model-1: Adjusted for calciphylaxis, age, ethnicity, diabetes mellitus and myocardial infarction

Multivariate Model-2: Adjusted for calciphylaxis, age, ethnicity, diabetes mellitus and warfarin use

Multivariate Model-3: Adjusted for calciphylaxis, age, ethnicity, diabetes mellitus, haemoglobin and albumin

## Discussion

Our data provides an overview of the natural history, current management strategies and outcomes of end- stage renal disease patients with calciphylaxis in the UK. The UKCS had a predominance of female and Caucasian distribution which is in agreement with previous observations, although the aetiology behind this association is still unexplained [16]. A high prevalence of diabetes and the higher median BMI noted in the UKCS patients were similar to that reported by Nigwekar et al. [17]. Both these findings support the possible link between calciphylaxis and the metabolic syndrome [18]. In addition, obesity and the distribution of skin lesions predominantly in adipose tissue rich areas (abdomen and thighs) noted in our cohort, is concordant with the mechanisms postulated for the development of CUA including increased arteriolar tensile stress and reduced local blood flow [19]. The diagnosis of calciphylaxis can be challenging and a vigilant clinical acumen is warranted to make a prompt diagnosis. The role of skin biopsy in the diagnosis of CUA is debatable [20]. A significantly higher c-reactive protein and a lower albumin and haemoglobin observed at calciphylaxis diagnosis in the UKCS is likely a reflection of the inflammatory nature of the condition. Inflammation is likely to be caused by calciphylaxis but may also contribute to the development of it; inflammatory mediators have been shown to induce vascular calcification in animal models and systemic calcification is also seen in inflammatory conditions [21–23]. Although extremely high, the mortality rate (72 deaths per hundred patient-years) and the median survival time (4 months) in the overall cohort is actually improved compared to that reported by Weenig et al. [3], probably attributed to a better understanding of the condition over the years and the multifaceted approach in the management. The devastating mortality of the condition is also highlighted in the propensity matched analysis of the 62 affected haemodialysis patients with annual mortality 67% versus only 10.2% in unaffected haemodialysis patients. Our study shows that the CUA wounds are slow to heal (median healing time (MHT)- 8 months), in keeping with the French (MHT- 6.4 months) and Australian registries (MHT- 8 months) [10, 24]. Patients in the UKCS group were more likely to be receiving calcimimetics and vitamin K antagonist, similar to other registry studies [17]. Warfarin inhibits the vitamin K–dependent carboxylation of the vascular calcification inhibitor, matrix-Gla protein thereby also providing biological plausibility to the hypothesis that vitamin K antagonists increase the risk of CUA [25]. These data have unsurprisingly generated significant interest in the use of vitamin K as a treatment for calciphylaxis. The use of vitamin K in our cohort was limited, therefore we are unable to draw any meaningful conclusions, however, we await the publication of Nigwekar et al’s randomised controlled trial which investigates the impact of phytomenadione on wound healing and all-cause mortality in 26 patients with CUA (NCT02278692).

Uncontrolled secondary hyperparathyroidism (SHPT) has been associated with an increased risk of calciphylaxis. The increased use of cinacalcet at baseline (19%) in the UKCS cohort may therefore represent underlying SHPT rather than a causative association. Deen et al. have suggested the beneficial effects of cinacalcet as a treatment for CUA in a recent review [26]. The EVOLVE study also suggested that patients receiving cinacalcet for SHPT were less likely to develop calciphylaxis; 18 patients assigned to placebo developed calciphylaxis versus six assigned to cinacalcet [27].

Sodium thiosulphate (STS) is widely used in the management of CUA, but a significant benefit with its use was not observed in our cohort. In a recent systematic review of 45 studies, sodium thiosulphate was identified as a potentially promising treatment in the management of CUA, however, its efficacy in modifying outcomes such as wound healing and pain is yet to be proven [8]. There are currently two randomised clinical trials (RCT) listed that are investigating the use of STS in calciphylaxis (ISRCTN73380053 and NCT03150420) [28]. To date, no single approach has been shown to be significantly beneficial in altering outcome and the approach in management in the UKCS has been multifaceted similar to that observed in other cohorts [29]. Due to the rarity and detrimental nature of CUA, obtaining RCT evidence on the management of this condition has been a challenge and identification of the optimal treatment strategy remains elusive but interest from commercial and academic groups has increased recently. SNF472 is an intravenous formulation of myoinositol hexaphosphate that can inhibit the formation and growth of hydroxyapatite crystals. It has been shown to attenuate vascular calcification in haemodialysis patients (CaLIPSO study) [30]. A small Phase 2 open-label study investigation suggested a potential benefit of SNF472 in calciphylaxis and a Phase 3 multi-centre study is currently in progress (NCT04195906) [31, 32]. Until evidence is available from RCT, the management strategy for CUA should essentially be a multifaceted approach which includes medical modification of risk factors, wound care, pain management, surgery and advanced care planning [33].

Both univariable and multivariable cox-regression analysis have shown calciphylaxis as a strong and independent risk factor associated with all-cause mortality. The hazard ratio for the presence of calciphylaxis observed in our univariable model is similar to that observed by Mazhar et al. in 2001 (HR:7.29; CI 2.88–18.5; p < 0.001), although the impact of cardiovascular disease events was not demonstrated in their models [34]. We found that vitamin K antagonist use, high c-reactive protein, low albumin and low haemoglobin in association with CUA were markers of poor prognosis similar to observations from the French cohort [24]. The role of iron overload on the pathogenesis of CUA is debatable. Farah et al. have shown an association between iron exposure and iron deposition in the skin biopsies of patients with calciphylaxis. The authors hypothesise that iron deposition may not be causative but may create a favourable milieu. In contrast to this observational data, the prospective, phase 3 randomised controlled trial of intravenous high dose iron versus low dose iron (PIVOTAL) in haemodialysis patients did not support this hypothesis, with no increased skin or subcutaneous-tissue disorders noted in the pro-active arm of the study [35, 36].

Our study is limited by the observational nature of the study methodology. The absence of cause of death data has limited our ability to identify the exact influence of calciphylaxis as a cause for mortality in the cohort. However, the study is strengthened by the well-structured registry-based data and a propensity matched comparative analysis with robust data from a dialysis cohort. Also, our study is novel in demonstrating the cardiovascular events history in the regression models showing association between calciphylaxis and all-cause mortality.

In conclusion, the UKCS endorses the findings from other registries that calciphylaxis has a very high mortality rate. This study has shown that calciphylaxis is a strong and independent risk factor associated with all-cause mortality. In addition, this study highlights that calciphylaxis lesions are slow to heal, if at all, and that no specific treatments have been associated with an improvement in outcomes. We await the outcome of future clinical trials to provide evidence-based treatment strategies. Until then a multifaceted, patient centred approach is appropriate for the management of this devastating and challenging condition.

## Electronic supplementary material

Below is the link to the electronic supplementary material.

Supplementary file1 (DOCX 21 KB)

Supplementary file2 (DOCX 83 KB)

Supplementary file3 (DOCX 16 KB)

## Data Availability

The datasets used and/or analysed during the current study are available from the corresponding author on reasonable request.
