# Supplementary material for: Calciphylaxis in end-stage kidney disease: outcome data from the United Kingdom Calciphylaxis Study
Source: J Nephrol. 2021 Feb 6;34(5):1537–45. doi: 10.1007/s40620-020-00908-9 (PMC8494680; doi:10.1007/s40620-020-00908-9)

**Supplementary fig. 1 Distribution of propensity scores after matching (Treatment unit-Calciphylaxis cohort, Control unit-Chronic Renal Insufficiency Standards Implementation Study-Haemodialysis cohort)**


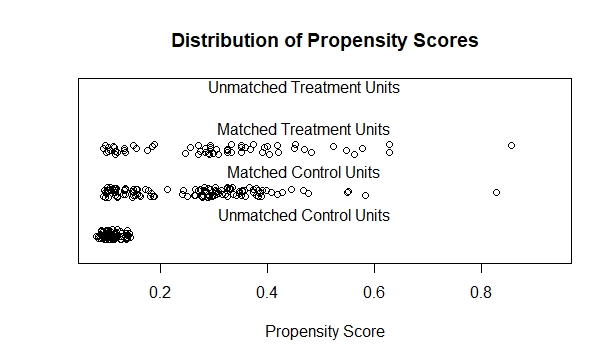

Supplement: Supplementary file 2 — Supplementary file2 (DOCX 83 KB) [file 40620_2020_908_MOESM2_ESM.docx]
