# Supplementary material for: Calciphylaxis in end-stage kidney disease: outcome data from the United Kingdom Calciphylaxis Study
Source: J Nephrol. 2021 Feb 6;34(5):1537–45. doi: 10.1007/s40620-020-00908-9 (PMC8494680; doi:10.1007/s40620-020-00908-9)
Supplement: Supplementary file 3 — Supplementary file3 (DOCX 16 KB) [file 40620_2020_908_MOESM3_ESM.docx]

**Supplementary table 1 Comparison of baseline characteristics (risk factors) with wound healing**

| **Variable** | **Healed (15)** | **Not healed (74)** | **p-Value** |
| --- | --- | --- | --- |
| Age, years | 63 (57-72) | 58.5 (53-69) | 0.21 |
| Gender, female | 10 (66.6%) | 44 (59.4%) | 0.60 |
| Ethnicity, Caucasian | 14 (93.3%) | 70 (94.6%) | 0.85 |
| Body mass index, (Kg/m^2^) | 34 (31-39) | 30.5 (25-38) | 0.31 |
| **Renal status at diagnosis**  Non-dialysis chronic kidney disease  Haemodialysis  Peritoneal dialysis | 2 (13.3%)  9 (60%)  4 (26.6%) | 9 (12.2%)  53 (71.6%)  12 (16.2%) | 0.60 |
| **Co-morbidities**  Ischaemic heart disease  Myocardial infarction  Cerebrovascular disease  Peripheral vascular disease  Diabetes mellitus  Bone fractures  Hypertension  Parathyroidectomy | 7 (46.6%)  2 (13.3%)  0  2 (13.3%)  8 (53.3%)  0  7 (46.6%)  1 (6.66%) | 28 (37.8%)  18 (24.3%)  12 (16.2%)  12 (16.2%)  40 (54.1%)  2 (2.7%)  51 (68.9%)  7 (9.5%) | 0.52  0.35  0.09  0.78  0.96  0.52  0.10  0.73 |
| **Medications**  Vitamin D analogues  Calcium based phosphate binders  Calcimimetics  Vitamin K antagonist (warfarin) | 10 (66.6%)  3 (20%)  5 (33.3%)  5 (33.3%) | 42 (56.7%)  16 (21.6%)  23 (31.1%)  30 (40.5%) | 0.48  0.89  0.86  0.60 |
| **Laboratory results**  Haemoglobin, g/L  Albumin, g/L  Corrected calcium, mmol/L  Phosphate, mmol/L  Alkaline phosphatase, units/L  Parathyroid hormone^a^, ng/L  C-reactive protein^b^, mg/L | 107 (97.5-116)  35 (31.5-37)  2.29 (2.24-2.58)  1.76 (1.18-1.99)  116 (83.5-182.5)  183 (55.5-393.5)  14 (4-25) | 92.5 (80-105)  26 (21-33)  2.39 (2.23-2.51)  1.64 (1.13-2.0)  172 (114-293)  439 (92-726)  81 (28-159) | **0.008**  **0.001**  1.00  0.835  0.073  0.266  **0.002** |

a-missing Parathyroid hormone levels in 32 patients, b-missing CRP in 7 patients

UKCS-United Kingdom calciphylaxis study. Categorical variables expressed as number (percentage) and continuous variables expressed as median (interquartile range)
